# Chitinous material bioconversion by three new chitinases from the yeast *Mestchnikowia pulcherrima*

**DOI:** 10.1186/s12934-024-02300-9

**Published:** 2024-01-20

**Authors:** Marina Minguet-Lobato, Fadia V. Cervantes, Noa Míguez, Francisco J. Plou, María Fernández-Lobato

**Affiliations:** 1 Department of Molecular Biology, Centre for Molecular Biology Severo Ochoa (CBMSO, CSIC-UAM), University Autonomous from Madrid, C/ Nicolás Cabrera, 1. Cantoblanco, Madrid, 28049 Spain; 2 Institute of Catalysis and Petrochemistry, CSIC. C/ Marie Curie, 2. Cantoblanco, Madrid, 28049 Spain

**Keywords:** *Mestchnikowia*, Chitinase, GH18, Chitin, Chitosan, Biotransformation, Chitin wastes, Chitooligosaccharides

## Abstract

**Background:**

Chitinases are widely distributed enzymes that perform the biotransformation of chitin, one of the most abundant polysaccharides on the biosphere, into useful value-added chitooligosaccharides (COS) with a wide variety of biotechnological applications in food, health, and agricultural fields. One of the most important group of enzymes involved in the degradation of chitin comprises the glycoside hydrolase family 18 (GH18), which harbours endo- and exo-enzymes that act synergistically to depolymerize chitin. The secretion of a chitinase activity from the ubiquitous yeast *Mestchnikowia pulcherrima* and their involvement in the post-harvest biological control of fungal pathogens was previously reported.

**Results:**

Three new chitinases from *M. pulcherrima*, MpChit35, MpChit38 and MpChit41, were molecularly characterized and extracellularly expressed in *Pichia pastoris* to about 91, 90 and 71 mU ml^− 1^, respectively. The three enzymes hydrolysed colloidal chitin with optimal activity at 45 ºC and pH 4.0-4.5, increased 2-times their activities using 1 mM of Mn^2+^ and hydrolysed different types of commercial chitosan. The partial separation and characterization of the complex COS mixtures produced from the hydrolysis of chitin and chitosan were achieved by a new anionic chromatography HPAEC-PAD method and mass spectrometry assays. An overview of the predicted structures of these proteins and their catalytic modes of action were also presented. Depicted their high sequence and structural homology, MpChit35 acted as an exo-chitinase producing di-acetyl-chitobiose from chitin while MpChit38 and MpChit41 both acted as endo-chitinases producing tri-acetyl-chitotriose as main final product.

**Conclusions:**

Three new chitinases from the yeast *M. pulcherrima* were molecularly characterized and their enzymatic and structural characteristics analysed. These enzymes transformed chitinous materials to fully and partially acetylated COS through different modes of splitting, which make them interesting biocatalysts for deeper structural-function studies on the challenging enzymatic conversion of chitin.

**Supplementary Information:**

The online version contains supplementary material available at 10.1186/s12934-024-02300-9.

## Background

Chitin is a water-insoluble linear homo-polysaccharide consisting of β- (1,4) linked N-acetyl-D-glucosamine units (GlcNAc). It is the second most abundant biopolymer in nature, after cellulose, as well as one of the major structural components of the exoskeleton of arthropods, and fungi cell walls [1, 2]. Besides of its plentiful natural occurrence, million tons of crustacean waste containing chitin (15–40%) are also generated by the shellfishery per year, that are directly discarded into landfills or returned to the oceans, creating a problem in the ecosystems. Thus, public policies and bioeconomy programs for the development of sustainable seafood systems as well as biorefinery pipelines for crustacean waste valorisation are emerging [3, 4]. The partial de-N-acetylation of chitin generates chitosan, a polymer of GlcNAc and D-glucosamine (GlcN) units. Both biopolymers possess suitable inherent biological and chemical properties, including biocompatibility, biodegradability, bio-adhesion ability, nontoxicity, and non-immunogenicity, so they can be used in a wide range of food, agriculture, and biomedical applications. Nevertheless, chitin shows a semi-crystalline structure with many intra- and intermolecular hydrogen bonds that causes it to be insoluble in most of the solvents, and chitosan is only soluble in weak acidic environments [3, 5–7]. The chitooligosaccharides (COS), obtained from depolymerization of both biopolymers, can be fully acetylated (*fa*COS), partially acetylated (*pa*COS), and fully deacetylated (*fd*COS). These water-soluble oligomers retain characteristics of the polymers from which they come, and are already being applied in different agricultural, biotechnology and biomedical fields [8–10]. COS can be chemically produced from chitin and chitosan using environmental unfriendly solutions, concentrated acids, or oxidative agents, which make difficult to control their degree of polymerization (DP), degree of acetylation (DA) and pattern of acetylation (PA). In this context, enzymes from microbial sources that act synergistically to degrade the chitinous biomass, such as chitinases, chitosanases and chitin de-acetylases among others, have generated an inherent interest as tools for a broad range of biotechnological applications, highlighting the production of value-added soluble derived oligomers in a safer and more controlled manner [11–13].

Chitinases are produced by wide type of microorganisms, plants, and animals. They are involved in a variety of processes, including remodelling of chitin in cell walls of fungi and exoskeleton of crustacean, defence mechanisms against pathogens, or the use of chitin as C/N carbon source. Most chitinases are glycosyl hydrolases (GH) classified into families 18, 19 and 20 (http://www.cazy.org) [14] that cleave at terminal or internal β- (1,4) glycosidic linkages of chitin chains. Enzymatic degradation of this polymer occurs from random internal bonds (endo-mechanism), or from the non-reducing or reducing chain ends (exo-mechanism), yielding mainly *fa*COS, di-acetyl chitobiose ((GlcNAc)_2_) or GlcNAc (this last by β-N-acetyl-hexosaminidases). The GH18 protein family use a substrate-assisted catalytic mechanism where the acid protonating the glycosidic bond to be hydrolysed is a conserved glutamate residue, in the conserved DxDxE catalytic sequence, and the nucleophile is the oxygen of the GlcNAc-N-acetyl group, the activity thus depending on acetyl groups of substrates. The overall structure of GH18 included an essential catalytic domain exhibiting a characteristic (β/α)8 TIM-barrel structure [15]. Some GH18 chitinases also contain an extra α + β insertion domain (CID) that was first observed in bacteria, so they are frequently named as bacterial-type chitinases, and those lacking this domain are named as plant-type chitinases. The structural characteristics of the first type have been extensively studied in the exo-chitinases SmChiA and SmChiB from *Serratia marcescens*, but also in the chitotriosidase HCHT from mammals. In all cases, these enzymes harbour a long and deep substrate-binding cleft, decorated with multiple aromatic residues [16–18]. In contrast to these tunnel-like grooves, a much shallower substrate-binding cleft is displayed in most of the structurally resolved plant-type chitinases, as in the endo-chitinases ScCTS1 from *Saccharomyces cerevisiae*, SmChiC2 from *S. marcescens* and ThChit33 from *Trichoderma harzianum* [19–21].

*Mestchnikowia pulcherrima* is an ubiquitous yeast that mainly colonizes fruits surfaces and flowers. The large number of extracellular hydrolytic enzymes expressed by this organism (amylases, cellulases, glucanases, β-glucosidases, etc.), some of which enhance the release of aromatic compounds, makes it of biotechnological interest, especially for the wine industry. Moreover, *M. pulcherrima* is already used as biological control agent due to the production of the red pigment pulcherrimin that shows antifungal activity [22–24]. Concerning chitinolytic enzymes, the secretion of a chitinase activity from *M. pulcherrima* MACH1 and their involvement in the post-harvest biological control of *Botritys cinerea*, a fungal pathogen affecting many important agricultural crops, was previously reported [25]. The exposition to *Penicillium digitatum* also caused the expression of a chitinase from *Mestchnikowia fructicola*, and it was speculated that this enzyme could be involved in the degradation of the pathogen cell-wall. In fact, the chitinase MfChi from *M. fructicola* was already expressed in *Pichia pastoris* and its antifungal activity proved [26, 27]. In this study, three chitinases from *M. pulcherrima* were molecularly characterized, successfully expressed in *P. pastoris* and their activities over different chitinolytic materials analysed using new anionic HPAEC-PAD techniques. The overall structure of these proteins was also predicted.

## Results and discussion

### Molecular characterization and heterologous expression of three chitinases from *Mestchnikowia pulcherrima*

**Fig. 1:**
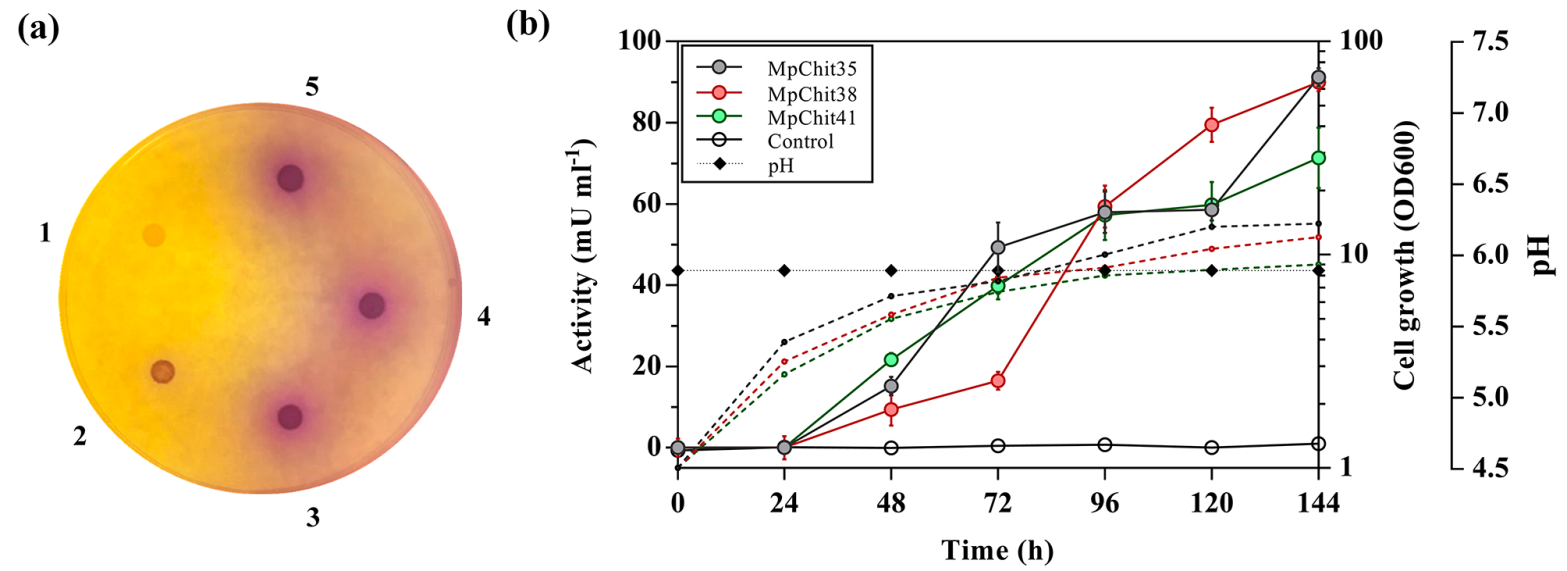
Analysis of chitinase activity on plate and heterologous expression of the *M. pulcherrima* chitinases. **(a)** Chitinolytic activity assay on solid BPC medium of *P. pastoris* (1) as negative control, *M. pulcherrima* (2), and transformants of *P. pastoris* expressing chitinases MpChit35 (3), MpChit38 (4) and MpChit41 (5). **(b)** Activity profiles of *P. pastoris* cultures expressing the referred chitinases from *M. pulcherrima* after methanol induction during the indicated times. Cell growth (OD600, dashed lines), pH (diamonds) and extracellular chitinase activity (solid lines) of the transformants expressing the referred proteins using colloidal chitin as substrate. Activity of *P. pastoris* culture including pIB4 vector was used as negative control (empty circles). Each point of activity represents the average of three independent measurements and standards errors are indicated

At the beginning of this work, the chitinase activity of the yeast *M. pulcherrima* IFI 1205 was verified on plates containing colloidal chitin (CC) as carbon source and bromocresol purple. This pH indicator changed the color of the medium including/surrounding the yeast, from the initial yellow to purple, as result of the alkalinization occurring after the polysaccharide hydrolysis [28]. *Pichia pastoris* was used in this assay as a negative control, without detecting any cellular color change (Fig. 1a). The genome of *M. pulcherrima* IFI 1205 has not been sequenced, but that of *M. pulcherrima* APC1.2 has already been annotated [22] (GenBank Accession Number: GCA_004217705.1), and at least three coding sequences (CDS) responsible for potential GH18 chitinases were identified. Pairs of primers were designed based on the functional annotation of *M. pulcherrima* APC 1.2 genome (Additional file 1: Table S1) and used in PCR reactions together with *M. pulcherrima* IFI 1205 genomic DNA, which allowed us to amplify the three possible chitinase CDS of this last organism. The amplified DNA fragment sizes were 1017, 1083 and 1092 bp, showed 96.60%, 96.3% and 96.15% of identity with the *M. pulcherrima* APC1.2 annotated CDSs, and codified for potential proteins of 338, 360 and 363 amino acids, respectively. The deduced amino acid sequences of the first two proteins showed a putative signal peptide, with a protease SPase I cleavage between residues 19 and 20 (with a probability of 0.983 and 0.975, respectively), whereas no signal peptide was predicted for the third one (Additional File 1: Figures S1 and S2). The lack of signal peptide could suggest the involvement of MpChit41 in processes related to cell wall morphogenesis or yeast division.

Based on their sequences, a theoretical molecular weight of about 35, 38 and 41 kDa was estimated for the potential chitinases that were named MpChit35, MpChit38 and MpChit41, respectively. The three CDS were cloned in the plasmid pIB4 fused to the MFα secretion signal and integrated into the *P. pastoris* genome, where their expression was controlled by the *AOX1* promoter, and therefore by methanol. The functionality of the sequences analysed was clearly demonstrated in the heterologous system since the obtained transformants were surrounded by a wide halo of chitin hydrolysis on CC and bromocresol purple plates (Fig. 1a). The strategy used in this work for obtaining the *P. pastoris* transformants was already used to successfully express the chitinases ThChit33 and ThChit42 from the fungi *T. harzianum* [29, 30], and besides to prove the antifungal activity of the chitinase MfChi from *M. fructicola* [27].

Chitinolytic activity of the *P. pastoris* transformants expressing the chitinases from *M. pulcherrima* was also evaluated in a methanol-base liquid medium. This activity was detected in the yeast extracellular media and the highest values obtained were 91.2 (for MpChit35), 89.6 (for MpChit38) and 70.8 (for MpChit41) mU ml^− 1^. As expected, the yeast transformant including the empty vector pIB4, used as negative control, did not show any hydrolytic activity over CC (Fig. 1b). Thus, we achieved for the first time the heterologous expression of three new active chitinolytic enzymes from *M. pulcherrima* with good expression levels.

### Sequence and structural analysis of the chitinases from *M. pulcherrima*

Multiple sequence alignments (MSAs) were performed with the amino acidic sequences of the three proteins from *M. pulcherrima* and homologous chitinases (Additional File 1: Figures S1 and S2). MpChit35 and MpChit38 showed close homology between them (78.4%) and to the plant-type endo-chitinase ScCTS1 from *S. cerevisiae* (52.5 and 48.4%, respectively). These sequences also revealed homology to fungal plant-type endo-chitinases AfChiA1 from *Aspergillus fumigatus* (35.5 and 34.0%, respectively) and ThChit33 from *T. harzianum* (40.3 and 35.8%, respectively), as well as the plant enzymes HevA from *Hevea brasiliensis* (35.7 and 34.2%, respectively) and PgChi from *Punicca granatun* (36.0 and 33.0%, respectively). However, MpChit41 revealed homology to some fungal bacterial-type exo-chitinases such as AfChiB1 from *A. fumigatus* (36.0%) and ThChit42 from *T. harzianum* (32.7%) and the endo-chitinase CiChi1 from *Coccidioides immitis* (33.8%). MSA also included the bacterial exo-chitinases SmChiA from *S. marcescens* (27.4%), BcChiA1 from *Bacillus circulans* (30.2%) and the hyper transglycosylating endo-chitinase SpChiD from *Serratia proteamaculas* (28.0%).

**Fig. 2:**
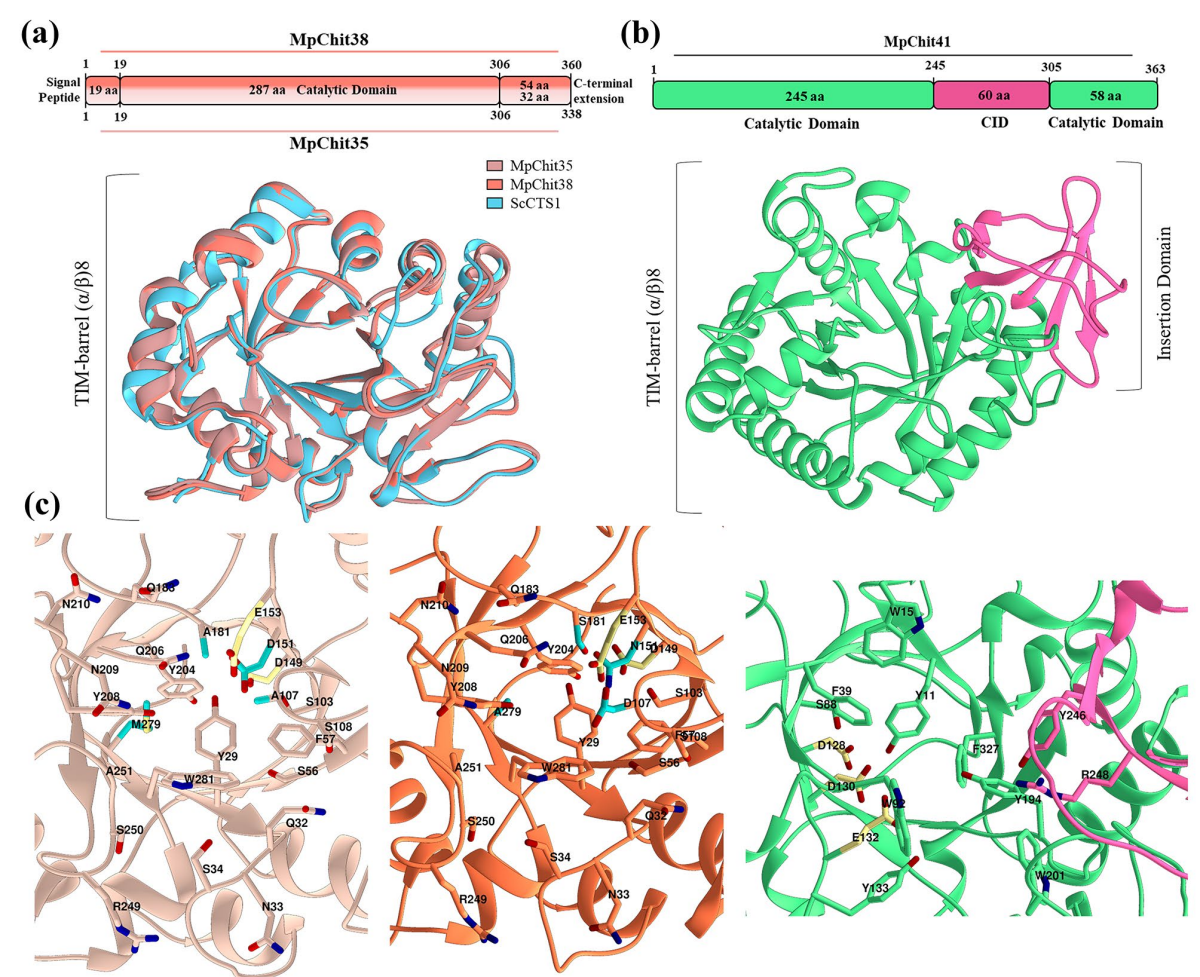
Structural models of the three *M. pulcherrima* chitinases. **(a)** Linear scheme of the MpChit35 and MpChit38 sequences (top) and superimposition of their overall predicted catalytic domains structures with ScCTS1 from *S. cerevisiae*. **(b)** Linear schema of the MpChit41 sequence (top) and its overall predicted structure. **(c)** Substrate-binding grooves of chitinases MpChit35, MpChit38 and MpChit41 (from left to right). Potential residues at catalytic groove of each chitinase are indicated and numbered. Catalytic residues in DxDxE sequence of the three chitinases are marked in yellow. Residues at the same position that are different between chitinases MpChit35 and MpChit38 at their catalytic grooves are indicated in blue

The deep learning-based approach AlphaFold was used to construct the three-dimensional (3D) structure models of the chitinases from *M. pulcherrima*. Most of the residues with very low scores (pDDLT < 50, per-residue confidence score) were located at C-terminal regions of MpChit35 and MpChit38. These regions lacked any tertiary structure on the 3D generated models. In fact, C-terminal sequences largely overlap with consensus intrinsically disordered regions (IDRs) (Additional File 1: Figure S1). Most of the low and very low confidence protein regions of the well-defined 3D structures that DeepMind’s algorithm failed to predict are likely to be intrinsically disordered. These regions are thought to acquire concrete structures dependent on the context and to participate in coupled folding, binding and dynamic processes determining protein functions [31]. Taken these considerations together, valid structural models of these chitinases were obtained (Fig. 2).

A visual inspection together with MSAs allowed us to highlight those amino acids that could be involved in catalysis (Fig. 2c), as was previously reported that surrounding amino acids of catalytic residues participate in catalysis, even when the distances are far [32]. The general structural features of chitinases GH18 were displayed in all these models. The occurrence of the conserved TIM-barrel fold (eight parallel β-strands alternated with eight α-helix topology), conforming the catalytic domain, was common for all the referred enzymes (Fig. 2a, b). The GH18 chitinase catalytic signature (DxDxE) was conserved in MpChit35 (Asp149, Asp151, Glu153) and MpChit41 (Asp128, Asp130, Glu132), both located at the end of the predicted β4 strand of the barrel (Fig. 2c). However, in MpChit38 the second Asp on the catalytic sequence was replaced by a non-conserved Asn151, constituting an unusual sequence DxNxE for an active wild-type enzyme (Additional File 1: Figure S1), but that was previously reported in mutagenic chitinase variants showing partial removal of their hydrolytic activity [33, 34]. The substrate binding GH18 sequence (SxGG) was also conserved in the three new chitinases, that was followed by Ala107 in MpChit35, Asp107 in MpChit38 and Trp92 in MpChit41 (Additional File 1: Figures S1 and S2). Structural model of MpChit38 positioned Asp107 very close to the catalytic Glu153 (Fig. 2c). The sequence SxGG followed by Asp was also detected in the chitinase ThChit33 from *T. harzianum* (Asp117). This Asp is also structurally very close to the enzyme catalytic Glu, and is involved in the enzyme activity clamping the bended conformation of the substrate at catalytic groove [20]. Therefore, the possibility that Asp107 could be involved in the MpChit38 activity could not be eliminated either.

The six conserved cysteine residues that form the three disulphide bonds typically found in endo-chitinases such as ScCTS1 were also detected in MpChit35 and MpChit38 (Additional File 1: Figure S1) [19]. An additional α + β insertion (CID) domain composed by five antiparallel β-strands and a short α-helix between β7-strand and α7-helix of the TIM barrel was found in MpChit41 (Fig. 2b), increasing the depth of the substrate-binding cleft, in comparison of the apparently shallow catalytic groove of MpChit35 and MpChit38 (Fig. 2a, c). The YxR sequence (Tyr246 and Arg248) typically found on CID domains was also conserved in the protein of 41 kDa (Additional File 1: Figure S2) [35]. Whilst MpChit41 also displayed a large path of highly conserved solvent-exposed aromatic residues conforming the active-site groove classically found in exo-acting bacterial-type chitinases, a single highly conserved tryptophan was present in the MpChit35 and MpChit38 models (Trp281 in both cases), as typically occurs in the plant-type chitinases (Fig. 2c) [17, 19, 36–38].

### Protein and enzymatic characterization of MpChit35, MpChit38 and MpChit41

**Fig. 3:**
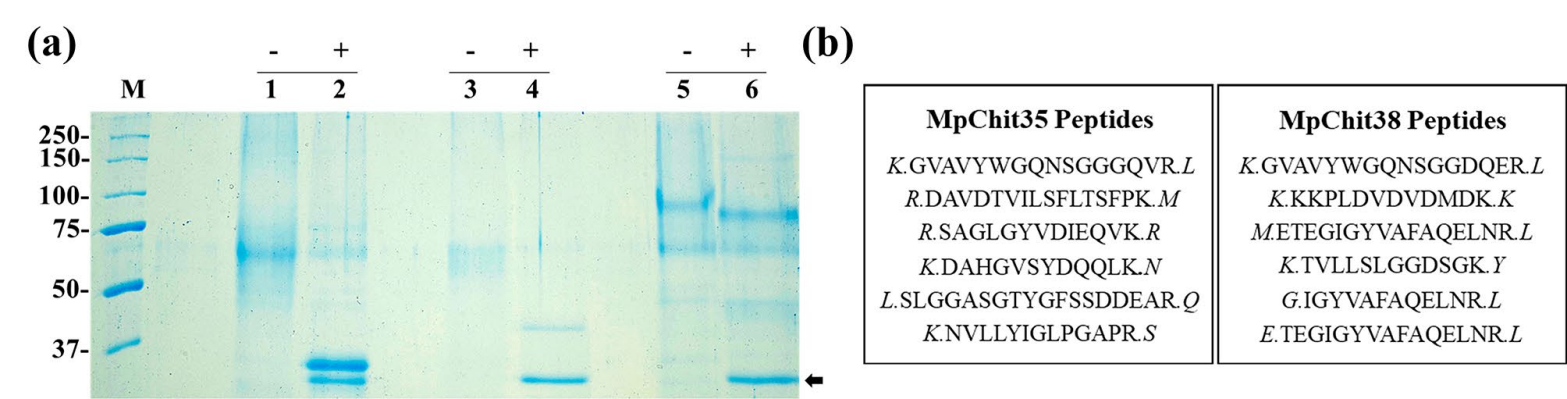
Analysis of the chitinases glycosylation on SDS-PAGE. **(a)** MpChit35 (lines 1 and 2), MpChit38 (lines 3 and 4) and MpChit41 (lines 5 and 6) before (-) and after (+) 24 h digestion with PNGase F (black arrow, 36 kDa). Samples were prepared from concentrated 144 h culture filtrates. Numbers on the left indicate the positions of molecular mass standards (lane M) in kDa. **(b)** Representative peptides identified by RP-LC-MS/MS of the indicated chitinases in the protein bans of about 36 kD (line 2) and 38 kDa (line 4) of panel (**a**)

Extracellular proteins produced by *P. pastoris* cells transformed with the pIB4 derivative construction were analysed by SDS-PAGE gels. Although the theoretical molecular weight of MpChit35, MpChit38 and MpChit41 were about 35, 38 and 41 kDa, respectively, the three proteins were expressed in the heterologous system as glycoproteins of higher molecular weights (Fig. 3a). Thus, apparently MpChit35 and MpChit38 migrated as majority protein bands of about 65–70 kDa, and MpChit41 as two bands of about 70 and 100 kDa, all with high molecular weight smears, most visible in the first one, which are characteristic of glycosylated proteins. In fact, six potential *N*-glycosylation sites were predicted in the MpChit35 sequence, eight in MpChit38 and seven in MpChit41 (Additional File 1: Figures S1 and S2). The three proteins were treated with PNGase F, an amidase that remove N-linked oligosaccharides from glycoproteins by cleaving between the innermost GlcNAc and Asn residues [39], with different results. Thus, while MpChit35 and MpChit38 were almost completely deglycosylated since protein bands of the expected theorical weight were visualized, treatment with PNGase F poorly altered the electrophoretic profile of MpChit41 that apparently only reduced the size of the 100 kDa band to about 85–90 kDa (Fig. 3a). Four potential O-glycosylation sites were predicted in the sequence of MpChit41 (Ser2, Ser254, Ser265 and Thr266), so it most likely also presents this type of modification.

Deglycosylation of chitinases MpChit35 and MpChit38 was verified by in situ trypsin digestion of the protein bands obtained after PNGase F treatment, of about 35 and 38 kDa, respectively, followed by reverse phase HPLC coupled to mass spectrometry (RP-LC-MS/MS). Both protein bands retrieved peptides located in the sequence of the analysed chitinases (Fig. 3b) revealing their identity. The 100 kDa band corresponding to MpChit41 was also subjected to this analysis but without any result, possibly due to the high degree of glycosylation of the sample.

**Fig. 4:**
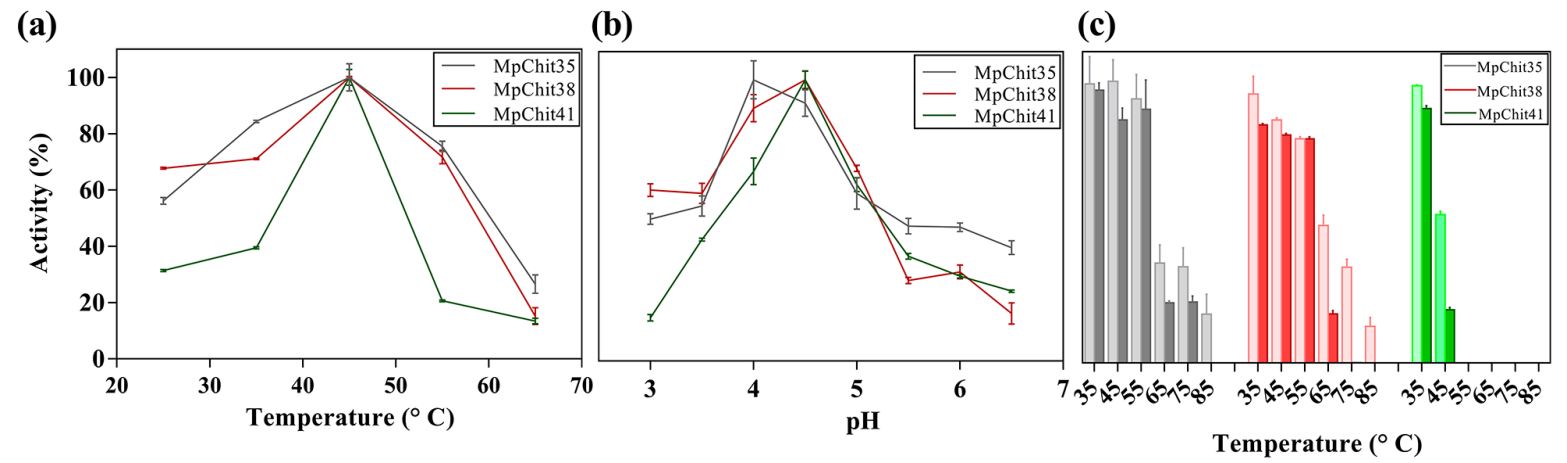
Effect of pH and temperature on hydrolytic activity of chitinases from *M. pulcherrima*. Optimal temperature **(a)** and pH **(b)** for the CC hydrolysis was evaluated. Activities after 30 (clear bars) and 60 min (dark bars) of incubation **(c)** at the referred temperatures. Percentages are relative to maximum activity values in **(a)** and **(b)** and to proteins not incubated at the indicated temperatures in **(c)**. Data are the average of three measures and standards errors are shown

The hydrolytic activities of the three native chitinases were evaluated to characterise the optimal conditions for enzymatic assays using CC as substrate. All three exhibited its maximum activity at about 45 ºC (Fig. 4a), as did chitinases SpChiD, CsChiL, ThChit33 and EcChi1 [29, 40–42], and pH 4.0-4.5 (Fig. 4b). Noticeably, MpChit35 and MpChit38 maintained up to 50% of their activities at pH 3.0 and 3.5, behaving like acidic chitinases, as occurred with the enzyme ScCTS1, whose unusual low optimal pH was speculated to be due to the presence of a conserved Asn215 at its catalytic groove [19]. This residue is also present in the two chitinases of *M. pulcherrima* (Additional File 1: Figure S1) and also in the SmChiB Asp215Asn variant, which present better activity in acidic pH than the wild type protein [32]. The three chitinases from *M. pulcherrima* were also incubated without substrate in the range from 35 to 85 °C for 30 and 60 min and their hydrolytic activity measured. MpChit35 and MpChit38 maintained 50% of their initial activity at 35–55 °C; but MpChit41 was more sensitive to temperature, as after 30 min at 45 °C its activity was decreased in almost 50% (Fig. 4c).

The three chitinases from *M. pulcherrima* expressed in *P. pastoris* released reducing sugars from chitinolytic materials of different sizes and DA, being CC their preferred substrate (Table 1). All also preferred the more acetylated chitosan as showed the higher hydrolytic activity on chitosan CHIT50.2 than on CHIT50.1, the first showing lower degree of deacetylation (DD) and therefore higher DA. A priori predictable data because the substrate-assisted catalytic mechanism of chitinases GH18 necessarily require a N-acetyl group (of GlcNAc) at position − 1 (cleave occurring between sugars − 1 and + 1) of the substrate being hydrolysed. Which means that the lower the DD, the greater substrate hydrolysis points and therefore the greater enzymatic activity [43–46].

**Table 1:** Substrate specificity of chitinases from *M. pulcherrima*

		Relative Activity (%)		
Chitinous Material	MW (kDa); DD (%)	MpChit35	MpChit38	MpChit41
Colloidal chitin	n.d; ≤ 5	100.0 ± 10.0	100.0 ± 11.2	100.0 ± 8.0
Chitosan CHIT50.1	75–125; ≥ 92	16.0 ± 2.0	14.0 ± 3.1	20.0 ± 5.3
Chitosan CHIT50.2	50–190; ≥ 75	72.2 ± 6.3	50.0 ± 2.4	72.0 ± 4.1
Chitosan CHIT100	100–300; ≥ 92	5.1 ± 1.2	n.ad.	n.ad.
Chitosan CHIT600	100–300; ≥ 92	10.0 ± 3.4	n.ad.	n.ad.

Values are the percentages of the maximum activity assayed for each enzyme type; activity data are average of 3 assays and standard errors are indicated; 100% activity: 0.4 U ml^− 1^, 0.5 U ml^− 1^ and 0.7 U ml^− 1^ for MpChit35, MpChit38 and MpChit41, respectively; DD, deacetylation degree; n.d., not determined; n.ad., no activity detected

Apparent kinetic parameters of these enzymes were determined with CC (Table 2). Michaelis-Menten fit was plotted for each enzyme (Additional File 1: Figure S3). Similar apparent *K*_m_ values were determined for MpChit35 and MpChit38, which were also quite similar of that obtained with the chitinase CsChiL (13.9 µg µl^− 1^) and EcChi1 (15.2 µg µl^− 1^) [40, 41]. However, MpChit41 displayed the highest *K*_m_ value, indicating that the two smallest chitinases from *M. pulcherrima* apparently show a higher affinity for this substrate than the larger one. In any case, the affinity of any of these chitinases for chitin was higher than that reported for SpChiD (83.3 µg µl^− 1^) [42]. Nevertheless, a priori the apparent catalytic efficiency (defined by the *k*_cat_/*K*_m_ ratio) showed that MpChit35 hydrolysed CC at least 2-fold more efficiently than MpChit38 and MpChit41.

**Table 2:** Apparent kinetic parameters of the referred enzymes on colloidal chitin

	*K*_m_ (µg·µl^− 1^)	*k*_cat_ (s^− 1^)	*k*_cat_/*K*_m_ (µl s^− 1^ µg^− 1^)
**MpChit35**	25.3 ± 4.4	0.04 ± 0.007	0.002 ± 0.0003
**MpChit38**	19.3 ± 3.3	0.02 ± 0.001	0.001 ± 0.0001
**MpChit41**	44.0 ± 9.4	0.03 ± 0.005	0.0007 ± 0.0001

Apparent *k*_cat_ values for each enzyme were calculated from V_max_ considering their theoretic molecular masses: 34,890.15 Da (MpChit35), 38,330.86 Da (MpChit38) and 40,639.64 Da (MpChit41)

The addition of metal ions to the reaction mixtures of the three enzymes was also evaluated (Table 3). A noticeable 2-times increment of the three chitinolytic activities was achieved by using Mn^2+^. Co^2+^ notably increased the activity of MpChit38, and to a lesser extent that of MpChit41 and MpChit35. However, the rest of the metal ions used either did not drastically affect (MpChit35, MpChit41) or decreased (MpChit38) the activity of these enzymes.

**Table 3:** Effect of different metal ions on the chitinolytic activity of the referred enzymes

	Relative Activity (%)		
Metal Ions	MpChit35	MpChit38	MpChit41
**Control**	100.0 ± 3.2	100.0 ± 6.0	100.0 ± 5.4
**MgCl**_**2**_	108.1 ± 4.2	49.0 ± 8.2	130.0 ± 6.0
**MnCl**_**2**_	220.3 ± 6.2	235.0 ± 9.2	253.3 ± 9.2
**CoCl**_**2**_	126.0 ± 3.4	291.0 ± 9.0	165.0 ± 5.1
**NiCl**_**2**_	88.0 ± 5.0	46.1 ± 9.4	153.0 ± 5.4
**CaCl**_**2**_	86.0 ± 5.1	30.3 ± 5.4	118.3 ± 4.4
**NaCl**	104.1 ± 7.3	42.1 ± 4.0	126.0 ± 5.4
**KCl**	88.2 ± 4.0	57.0 ± 5.3	122.0 ± 8.0

Values are the percentages of the activities without metal ions for each enzyme. Activity data are average of 3 assays and standard errors are indicated; 100% activity values as referred in Table 1

### Characterization of products formed from natural chitinous biopolymers

**Fig. 5:**
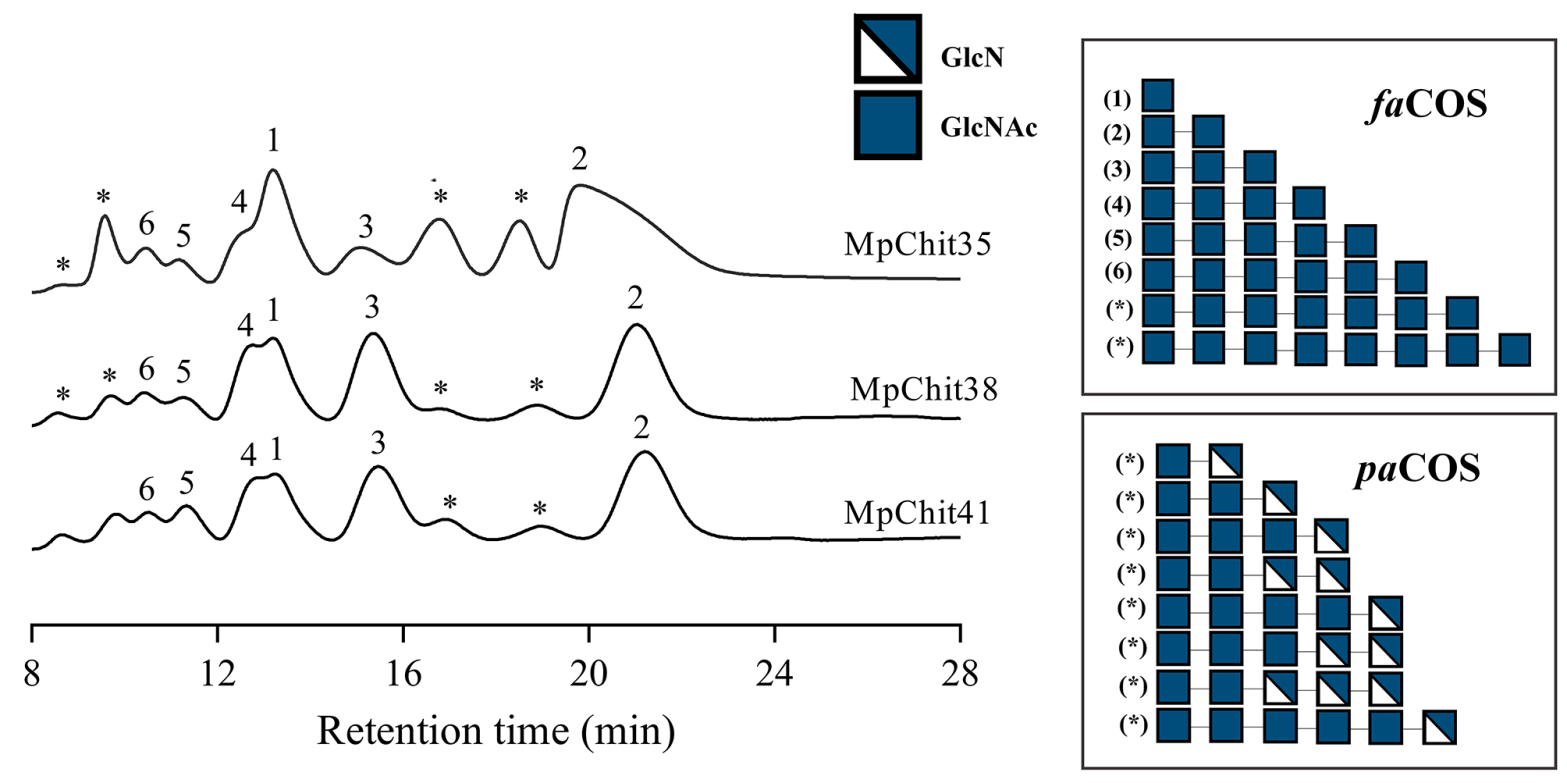
Representative HPAEC-PAD chromatographic profiles of the colloidal chitin degradation. Chromatograms of the 24 h reactions catalysed by the referred chitinases. CC was used as substrate. Peaks assignation: (1) GlcNAc, (2) (GlcNAc)_2_, (3) (GlcNAc)_3_, (4) (GlcNAc)_4_, (5) (GlcNAc)_5_, (6) (GlcNAc)_6_, (*) non-identified (probably *pa*COS). On the right a schematic representation of the polymerization degree and composition of reaction products predicted from MS data (Additional file 1: Table S2) is presented

HPAEC-PAD chromatography and mass spectrometry (MS) analyses were performed to analyse COS production from chitinous biopolymers. CC degradation with *M. pulcherrima* chitinases yielded product with molecular weights of *fa*COS (GlcNAc)_1−8_, as detected in MS spectra (Additional File 1: Table S2). In this work, we developed a new HPAEC-PAD method to analyse the activity of the studied chitinases using a PA-100 column that separated efficiently the main synthesized COS from the complex reaction mixtures. As typically occurs with these samples [13, 30], no correlation between the elution order and the COS degree of polymerization (DP) was achieved. Besides, and very likely because chitin was not initially 100% acetylated, masses corresponding to *pa*COS were also detected in the reaction mixtures, which were not identified in the chromatographic profiles by the lack of the corresponding standards (Fig. 5; Additional File 1: Table S2). In addition, the close elution of the monosaccharide GlcNAc and the tetrasaccharide (GlcNAc)_4_ compromised their quantification, but no their identification, as elution of the last occurs before the first one. In this context, it was possible to confirm from MpChit35 reactions that lower amounts of (GlcNAc)_4_ were obtained in comparison with GlcNAc, unlike MpChit38 and MpChit41 reactions, where signal corresponding to both products are very similar (Fig. 5). Furthermore, from the MS spectra, a low intensity of the (GlcNAc)_4_ mass was detected compared to that of GlcNAc in the MpChit35 reaction mixture. This contrasts with MpChit38 and MpChi41 reactions, where (GlcNAc)_4_ was one of the main masses detected, after that of (GlcNAc)_3_ (Additional File 1: Table S2).

**Fig. 6:**
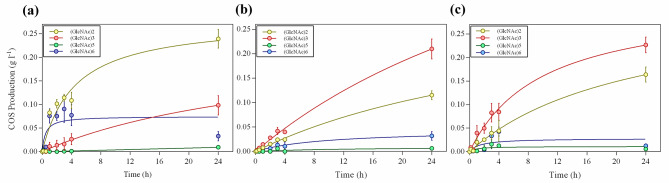
Product formation from colloidal chitin degradation. Time-course formation of quantifiable COS from CC in the MpChit35 (**a**), MpChit38 (**b**) and MpChit41 (**c**) reactions. Data are the average of two different measurements and standard errors are indicated

With the aid of the corresponding commercial standard, (GlcNAc)_2−3−5−6_, the products were identified and quantified (Fig. 6), confirming that (GlcNAc)_2_ was the major product generated by MpChit35, about 0.24 g l^− 1^, while that of MpChit38 and MpChit41 was (GlcNAc)_3,_ which produced about 0.21 g l^− 1^ and 0.23 g l^− 1^, respectively of these *fa*COS. Both enzymes also produced about 0.11–0.16 g l^− 1^ of (GlcNAc)_2_. Clear-cut yield of (GlcNAc)_3_ was also detected in the MpChit35 24 h-reaction (about 0.11 g l^− 1^), which correlated with the MS data (Additional File 1: Table S2), and its concentration increased over all analysed times of reaction (Fig. 6a). These results point out that hydrolysis occurs with at least 4 linked units of GlcNAc for all the three chitinases. We also deduced from these results that formation of monosaccharides was not derived directly from degradation of di-/tri-saccharides in a β-hexosaminidase manner, as these products were not hydrolysed upon 24 h reaction. Nevertheless, different modes of action between MpChit35 and MpChit38/MpChit41 could be deduced from the obtained data. Thus, MpChit35 could be an exo-chitinase that produces mainly (GlcNAc)_2_, as it was the main *fa*COS in all the reaction times analysed. However, MpChit38 and MpChit41 clearly are endo-chitinases, as (GlcNAc)_3_ was their main degradation product (Fig. 6b, c) in all the reaction times analysed. In addition, very minor amounts of (GlcNAc)_5−6_ were also detected in all reactions.

From a structural point of view, the initial detected differences in the architecture of the substrate-binding grooves of the three chitinases correlated with the well-known endo- and exo-fashion activities commonly found into members of the plant-type (MpChit35 and MpChit38) and bacterial-type (MpChit41) chitinases, respectively. However, according to the majority product that they form from CC, MpChit35 and MpChit41 seem to display the contrary modes of action, and although MpChit35 and MpChit38 show high sequence homology (78.4%), the latter acts as an endo-chitinase, as mentioned before, emphasizing that the residue differences detected in the catalytic grooves control the activity of these enzymes, turning them into targets to control the degree of polymerization of enzymatic products.

Mass spectrometry analysis was performed for the characterization of COS mixtures obtained from degradation of two chitosan types, differing in its degree of acetylation (DA), after 24 h of reaction (Additional File 1: Table S3 and S4). As expected, p.a.COS were mostly found in chitosan reactions when using the three enzymes from *M pulcherrima*. The larger the DP of products obtained from hydrolysis of both polymers, the smaller their DA.

**Fig. 7:**
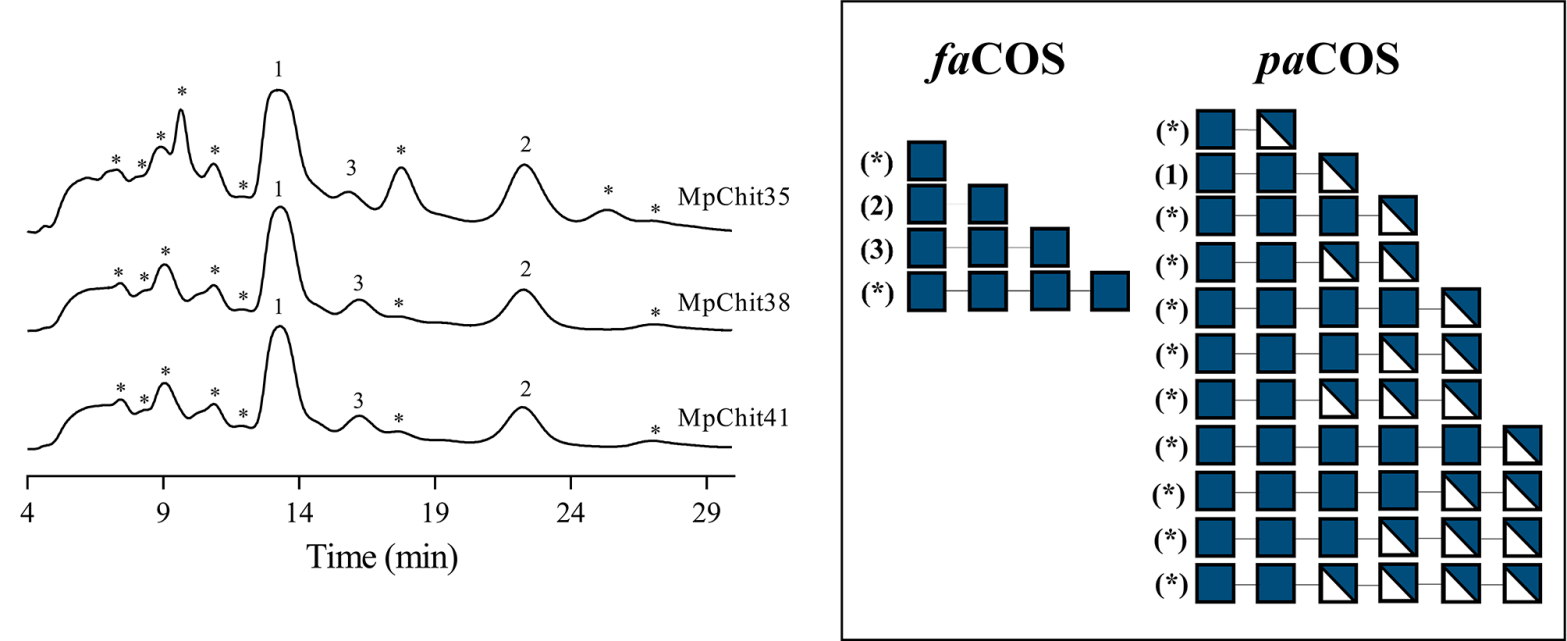
Representative HPAEC-PAD chromatographic profiles of the chitosan degradation. Chromatograms correspond to the COS mixtures obtained by hydrolysis of chitosan CHIT50.2 after 24 h. Peaks assignation: (1) apparently (GlcNAc)_2_-(GlcN), (2) (GlcNAc)_2_, (3) (GlcNAc)_3_, (*) non-identified. On the right a schematic representation of the polymerization degree and composition of reaction products predicted from MS data (Additional file 1: Table S4) is presented

HPAEC-PAD chromatography analysis was also performed with CHIT50.2, as the major relative activity after CC was obtained using this polymer (Table 1). Although many of the formed products could not be identified due to the unavailability of standards (Fig. 7), according to MS data (Additional file 1: Table S3, S4), the *pa*COS including 2 units of GlcNAc and one of GlcN ((GlcNAc)_2_-(GlcN), PA unknown) was the main product from both substrates analysed. This fact allows us to propose this *pa*COS as responsible of the main peak detected in the chromatographic profiles of the chitosan-based reactions (Fig. 7). Besides, mixtures obtained with MpChit38 and MpChit41 were also apparently enriched in (GlcNAc)_3_-(GlcN), being its PA also unknown. Interestingly, MpChit35 produced high mass intensities of (GlcNAc)_3_-(GlcN)_3_ from the two chitosan types. The enzymatic synthesis of the referred oligomer with alternating (GlcN)-(GlcNAc) units from *p*-nitrophenyl-diacetyl-chitobiose has been reported and its role as specific human chitotriosidase (HCHT) inhibitor demonstrated. The inhibition of this human enzyme has been speculated to be an important therapeutic target in systemic sclerosis [47].

Since the biological activities of COS are strongly influenced for its DP, DA and PA [11, 48, 49], the chitinase-catalysed synthesis becomes a powerful biotechnological strategy for the bioconversion of chitinous wastes to homogeneous mixtures of value-added *fa*COS and *pa*COS. In this context, the usage of specific and novel enzymes whose catalytic mechanisms are well-defined for the obtaining of different oligomers with also well-defined properties represents a sustainable alternative to traditional random chemical methods and expensive starting substrates. This work aims to described novel chitinolytic activities with structural peculiarities that transform chitinolytic polymers in different COS mixtures. Deeper structural-function studies should be accomplished to decipher the key features that determine the different action mode of these enzymes from *M. pulcherrima* and their functional improvement to increase or bias their biotechnological potential.

## Conclusions

In the present study, we successfully characterized for the first time an exo- and two endo-chitinases from *M. pulcherrima*, a yeast used as biological control agent against fungal phytopathogens, and we reported a new chromatographic method for the separation of the products generated from different chitinolytic materials. The presented 3D model analysis provides the structural details of the new proteins catalytic grooves and disclosed their interesting features regarding their different action modes, opening horizons for deeper structure-function studies of yeast chitinolytic systems. Unexplored partially acetylated COS mixtures were produced from chitosan using these enzymes, highlighting their biotechnological interest for the synthesis of high value-added products from industry chitinous wastes.

## Methods

### Chemicals

Chitin from shrimp shells (practical grade coarse flakes), chitosan CHIT50.1 (Product number: 900,344) and CHIT50.2 (Product number: 448,869), *N*-acetyl-*D*-glucosamine (GlcNAc) and biotin were from Sigma Aldrich (St. Louis, MO, USA). Chitosan from shrimp shells CHIT100 and CHIT600 were from Across Organics Chemicals (ThermoFisher Scientific, Waltham, Massachusetts, USA). Colloidal chitin (CC) was obtained as referred previously [30]. Basically, 10 g chitin in 10 M HCl was maintained 16 h at 4 ºC, filtered through glass thick fibres into ethanol and then chitin floccules were precipitated at 4 ºC, collected (5000x*g* during 10 min) and maintained in 0.1 M sodium acetate pH 4.0-4.5. Chitosan was dissolved in 0.1 M sodium acetate pH 4.0-4.5, in a final concentration of 10 g l^− 1^. Diacetyl-chitobiose ((GlcNAc)_2_), triacetyl-chitotriose ((GlcNAc)_3_), tetraacetyl-chitotetraose ((GlcNAc)_4_), pentaacetyl-chitopentaose ((GlcNAc)_5_) and hexaacetyl-chitohexaose ((GlcNAc)_6_) were from Megazyme (Bray, Irland). Nitrogen base w/o amino acids (YNB) was from Difco (BD, Sparks, MD, USA). Yeast extract, peptone, tryptone and agar were from Laboratorios Conda S.A. (Madrid, Spain).

### Microorganisms and culture conditions

*Metschnikowia pulcherrima* IFI 1205 (CECT 12827, Paterna, Valencia, Spain) was used in this study. *Escherichia coli* DH5α was used as host for DNA manipulations and *Pichia pastoris* (formally *Komagataella phaffii*) GS115 (*his4*-, Invitrogen, Carlsbad, CA, USA) as host for protein expression. Yeasts were maintained at 4 ºC on YEP solid medium (glucose 20 g l^− 1^, yeast extract 10 g l^− 1^, peptone 20 g l^− 1^, agar 20 g l^− 1^). The *P. pastoris* transformants were selected on MD medium (YNB 13.4 g l^− 1^, glucose 20 g l^− 1^, biotin 0.4 g l^− 1^). BMG and BMM media (both MD, 0.1 M potassium phosphate pH 6.0 and glycerol 4% or methanol 0.5%, respectively) were employed for transformant growing and protein expression analysis. A modified BPC medium (0.3 g l^− 1^ MgSO_4_ 7H_2_O, 3.0 g l^− 1^ (NH_4_)_2_SO_4_, 10.0 g l^− 1^ K_2_HPO_4_, 15 g l^− 1^ agar, 10 g l^− 1^ CC and 0.15 g l^− 1^ bromocresol purple, pH 4.7 adjusted with citric acid) was used for chitinase activity assays on plates (1% methanol was also used when *P. pastoris* transformant were screened) [28]. Yeasts were cultured at 30 ºC with orbital shaking (200 rpm), and growth was monitored spectrophotometrically at a wavelength of 600 nm (OD600).

### DNA amplification and cloning

Three putative chitinase coding sequences (CDS) from *M. pulcherrima* IFI 1205 were amplified from the genomic DNA of this organism, that was previously obtained using the procedure already described [50]. Basically, yeast cultured in YEP were re-suspended in SDS 3% (w/v), heated (55 °C for 15 min), kept on ice for 10 min in 3 M potassium acetate, and centrifuged at 4 °C (12100x*g*). DNA was precipitated by adding one volume of cooled isopropanol, recovered by centrifugation as before and treated with 20 mg l^− 1^ RNase (ThermoFisher, Waltham, Massachusetts, USA). Phusion High Fidelity DNA Polymerase (New England Biolabs, NEB, Herts, UK) and primers directed towards the CDS of 3 putative chitinases (Accession Number: QBM89584.1, QBM89583.1 and QBM86234.1) annotated in the genome of the *M. pulcherrima* strain APC 1.2 (GenBank Assembly Accession: GCA_004217705.1) were used. The potential CDS *mpchit35*, *mpchit38* and *mpchit41* (from the initial ATG to final TGA (*mpchit35, mpchit38*) or TAA (*mpchit41*)-stop codons) were obtained using the primer pairs CHI35F1-CHI35R1, CHI38F1-CHI38R1 and CHI41F1-CHI41R1, respectively (Additional file 1: Table S1). Taq II DNA Polymerase (New England Biolabs, NEB, Herts, UK) was used to add adenine nucleotides to the blunt-ends of the DNA fragments amplified. PCR products were purified from agarose gel (0.7%; /v) using NZYGelpure kit (NzyTech, Lisbon, Portugal), cloned into the pGEM-T Easy pre-linearized vector (Promega BioTech Ibérica, Alcobendas, Madrid) and verified by DNA sequencing (Macrogen, Madrid, Spain).

A derivative pIB4 (*HIS*4) vector [51], containing sequences for the mating factor-α (*MF*α) of *Saccharomyces cerevisiae* as secretion signal, and the methanol strong regulated alcohol oxidase 1 promoter (*AOX1*p), was used as expression vector. Initially, to clone the CDS of the 3 potential chitinases in this plasmid a restriction-free cloning procedure based in two PCR reactions was used [52], which only worked for *mpchit35* and *mpchit38*. First, the CDS of interest were amplified without their predicted signal peptides sequences, fused to short sequences complementary to sequences flanking the site of insertion in pIB4 using, in successful cases, primers CHI35F2 and CHI35R2 or CHI38F2 and CHI38R2 (Additional file 1: Table S1). PCR products were purified from agarose gels as above and used as mega-primers in the second PCR reaction using the initial pIB4 derivative vector as template. Final pIB4-mpCHIT35 and pIB4-mpCHIT38 constructions included the *mpchit35* and *mpchit38* CDS fused to *MF*α, respectively. Conditions for the first PCR reaction were: (i) 98 °C for 30 s; (ii) 30 cycles of 98 °C for 10 s, 60 °C for 30 s, 72 °C for 45 s; (iii) 72 °C for 600 s, and for the second PCR reaction, the same as the first, but with an annealing temperature of 72 °C and a final extension time of 210 s. In both, Phusion High Fidelity DNA Polymerase (New England Biolabs, NEB, Herts, UK) was used. To clone *mpchit41* CDS in pIB4, an in vivo assembly procedure involving recombination of two PCR amplicons was used [53]. Briefly, a first PCR to amplify the pIB4 vector using pIB4F and pIB4R primers, and a second PCR to obtain the *mpchit41* CDS flanked by the pIB4 recombination sequences using the CHI41F and CHI41R primers (Additional file 1: Table S1) were carried out. The PCR products were purified as before, mixed in a proportion 1(vector):5(insert) and transformed into *E. coli*, where performed the correct assembly of *mpchit41* in phase with *MF*α. Conditions for the first PCR reaction were: (i) 98 °C for 30 s; (ii) 30 cycles of 98 °C for 10 s, 62 °C for 30 s, 72 °C for 360 s; (iii) 72 °C for 600 s, and for the second PCR reaction, the same as the first, but with an annealing temperature of 60 °C and a final extension time of 45 s. Bacteria colonies including pIB4-MpCHIT38, pIB4-MpCHIT35 and pIB4-MpCHIT41 constructions were checked using universal AOX1 and AOX2 primers (Additional file 1: Table S1), DNA plasmids were extracted with NZYMiniprep kit (NzyTech, Lisbon, Portugal). Integrity of the constructions were verified by DNA sequencing (Macrogen, Madrid, Spain).

### *Pichia pastoris* transformation, protein expression and *N*-glycosylation analysis

The final pIB4 derivative constructions obtained in this work, were linearized with *Sal*I (New England Biolabs, NEB, Herts, UK) at locus *HIS4* and transformed into *P. pastoris* by electroporation according to the manual for protein expression in *Pichia* (Invitrogen, Carlsbad, CA, USA). Transformants of this yeast including the pIB4 empty vector was also obtained and used as control in the protein expression assays. Selected colonies were transferred from MD agar plates into 250 ml flaks containing 25 ml BMG and cultivated at 30 °C at 250 rpm until reaching log-phase growth (about 4–8 OD600). Cells were collected by centrifugation (1500x*g* 5 min), transferred to 1 L flaks containing 200 ml BMM and cultivated for 120 h. Induction of protein expression was conducted by adding 0.5% methanol every 24 h. The culture growth was periodically evaluated at 600 nm and their pH maintained (if required) using 1 M potassium phosphate pH 6.0. Cells were removed (1500x*g* 5 min), extracellular fractions filtered using 0.22 μm membranes (Merck Life Science, Madrid, Spain) and concentrated (if required) trough 30 (MpChit35, MpChit38) or 50 (MpChit41) kDa MWCO PES Filters (Sartorius, Madrid, Spain).

Proteins produced by the *P. pastoris* transformant including the pIB4 derivative constructions were evaluated in sodium dodecyl sulphate-polyacrylamide gels (SDS-PAGE, 12%) using BlueSafe stained (NzyTech, Lisbon, Portugal). No proteins were detected in the extracellular media of yeast transformed with the empty vector pIB4 [51]. Precision Plus Protein Standards Unstained 10–250 kDa (Bio-Rad, Madrid, Spain) were used as molecular weight markers. The protein levels were evaluated at 280 nm in a NanoDrop 1000 Spectrophotometer V3.8 (ThermoFisher Scientific Inc., Madrid, Spain) using bovine serum albumin as standard or in SDS-PAGE gels using the Image J Software Version 1.53t. Peptide-N-glycosidase F (PNGase F; New England Biolabs, NEB, Herts, UK) was used to evaluate the proteins (20 µg) glycosylation level. All reactions were carried out according to the manufacturer protocol and were analysed by SDS-PAGE.

### Proteomic analysis

Proteomic analysis was carried out in the CBMSO Protein Chemistry Facility that belongs to ProteoRed, PRB3-ISCIII. After drying, gel bands were faded in acetonitrile:water (1:1, v/v), reduced, alkylated, and digested in situ with trypsin (Promega Biotech Ibérica, Madrid, Spain) [54]. Digestion was stopped by the addition of 1% TA. Whole supernatants were dried down and then desalted onto ZipTip C18 Pipette tips (Merck Life Science, Madrid, Spain) until mass spectrometric analysis was performed by reverse phase-liquid chromatography (RP-LC-MS/MS) in an Easy-nLC 1200 system coupled to an ion trap LTQ-Orbitrap-Velos-Pro hybrid mass spectrometer (Thermo Scientific). The peptides were concentrated by reverse phase chromatography using a 0.1 mm × 20 mm C18 RP precolumn (Thermo Scientific), and then separated using a 0.075 mm x 250 mm C18 RP column (Phenomenex) operating at 0.25 µl min^− 1^. Peptides were eluted using the 60-min dual gradient: 5–25% solvent B for 45 min, 25–40% solvent B for 15 min, 40–100% solvent B for 2 min and 100% solvent B for 18 min (Solvent A: 0.1% formic acid in water, solvent B: 0.1% formic acid, 80% aetonitrile in water). ESI ionization was done using a Nano-bore emitters stainless steel ID 30 μm (Proxeon) interface at 2.1 kV spray voltage with S-Lens of 60%. The Orbitrap resolution was set at 30,000 [55]. Peptides were detected in survey scans from 400 to 1,600 amu (1 µscan), followed by 20 data dependent MS/MS scans (Top 20), using an isolation width of 2 u (in mass-to-charge ratio units), normalized collision energy of 35%, and dynamic exclusion applied during 60 s periods. Charge-state screening was enabled to reject unassigned and singly charged protonated ions. Peptide identification from raw data was carried out using PEAKS Studio Xpro search engine (Bioinformatics Solutions Inc., Waterloo, Ontario, Canada). False discovery rates (FDR) for peptide spectrum matches (PSM) was limited to 0.01. Only those proteins with at least two distinct peptides and at least one unique peptide being discovered from LC/MS/MS analyses were considered reliably identified.

### Chitinase activity

Unless otherwise indicated, chitinase activity was assayed colorimetrically, as reducing sugar release from CC and other substrates, by 3,5-dinitrosalicylic acid (DNS) method adapted to a 96-well microplate scale, described previously [30]. Reactions were carried out using 100 µl of enzyme solutions in a total reaction volume of 400 µl with 10 mg ml^− 1^ substrate in 0.1 M sodium acetate buffer. For specificity assays, polymeric substrates were used at 5 mg ml^− 1^. Reaction mixtures were incubated at 900 rpm in a VorTemp 56 Shaking Incubator (LabNet, Madrid, Spain) and stopped by boiling at 100 °C during 10 min. Remaining polysaccharides were precipitated by adding one volume of 0.2 M NaOH and removed by centrifugation (11000*xg*, 10 min). A standard curve of GlcNAc (0–3 g l^− 1^) was used. One unit of chitinase activity (U) was defined as that corresponding to the release of 1 µmol of reducing sugars per minute.

The pH and temperature dependence assays were performed using CC in 0.1 M sodium acetate buffer at the pH range 3.0–7.0 at 45 °C, and in the range of 25–65 °C, at pH 4.0 or 4.5. The thermostability assays were carried out by incubating enzymes at 35–85 °C during 30 and 60 min and then estimating their residual chitinase activity. To determine the metal ions effect on chitinase activity, reactions were performed in the presence of 1 mM of MgCl_2_, MnCl_2_, CoCl_2_, CaCl_2_, NaCl and KCl. Enzyme activities without metal ions addition were used as control.

For evaluation of chitinase activity on plates, the yeasts *M. pulcherrima* and *P. pastoris* were grown in 10 ml of YEP medium and transformants of *P. pastoris* in BMG medium to about 1 OD600. Then the yeast cultures were washed in 10 ml of water and a drop of 10 µl was added to plates of BPC medium that included CC and the pH indicator bromocresol purple. Plates were maintained 24 h at 30 ºC. Breakdown of chitin cause a shift in the pH towards alkalinity and thus a color change of the pH indicator from yellow to purple [28].

The apparent Michaelis-Menten kinetic constants were determined using 0.1–30 mg ml^− 1^ of CC at the optimal reaction conditions for each enzyme type. The plotting and analysis of the curves were carried out using GraphPad Prism 9 Software and kinetic parameters were calculated by fitting the initial rate values to the Michaelis-Menten equation. Based on their sequences, the theoretical molecular weights of proteins MpChit35, MpChit38 and MpChit41 were 34,890.15 Da, 38,330.86 Da and 40,639.64 Da, respectively, data that were used to calculate their molar concentrations. All reactions were performed in triplicate and standard errors were calculated.

### Characterization and quantification of polysaccharide degradation products by HPAEC-PAD and MALDI-TOF-MS

Reactions were performed as referred in a final volume of 4 ml, using 10 mg ml^− 1^ CC, chitosan CHIT50.1 or CHIT50.2 in the presence of 0.5–0.9 U ml^− 1^ of chitinases. To follow the progress of the enzymatic reactions, aliquots of 0.5 ml were taken at different reaction times, boiled during 10 min and analysed by HPAEC-PAD and MALDI-TOF-MS.

High performance anion-exchange chromatography coupled with pulsed amperometric detection analysis (HPAEC-PAD) was carried out on an ICS3000 system (Dionex, Thermo Fischer Scientific Inc., Waltham, MA, USA) consisting of a SP gradient pump, an electrochemical detector with a gold working electrode and Ag/AgCl as reference electrode and an autosampler (model AS-HV). All eluents were degassed by flushing with helium. An anion-exchange 4 × 250 mm Carbo-Pack PA-100 column (Dionex) connected to a 4 × 50 mm CarboPac PA-100 guard column was used at 30 °C. Eluent preparation was performed with MilliQ water, 50% (w/v) NaOH and sodium acetate trihydrate. The compounds were eluted with a gradient method using a flow rate of 0.8 ml min^− 1^ in which the initial mobile phase was 7.5 mM NaOH for 30 min, then from minute 30 to minute 35, the mobile phase was increased gradually until it reached a concentration of 120 mM sodium acetate and 20 mM NaOH, which was further maintained during 5 min. Finally, initial conditions were reset and maintained for 20 min to equilibrate the column. The chromatograms were analyzed using the Chromeleon software. Calibration curves were set for each commercially available *fa*COS standards (0.01–0.1 g l^− 1^), which were used for identification and quantification of the different peaks.

Hydrolysis product mixtures from chitinolytic material were analysed by matrix-assisted laser desorption ionization of flight mass spectrometry (MALDI-TOF-MS) after 24 h reaction, using a mass spectrometer with Ultraflex III TOF/TOF (Bruker, Billerica, MA, USA) and an NdYAG laser. Mixtures were first treated with high cationic exchange resin to minimize ionization inhibition. Registers were taken in positive reflector mode, within external calibration and 20 g l^− 1^ 2,5-dihydroxybenzoic in acetonitrile (3:7) as a matrix. Samples were mixed with the matrix in a 4:1 ratio and 0.5 µl were analysed. The acquisition range was from m/z 40 − 3,000 Da.

### Sequence analysis and molecular modeling

Sequences encoding chitinases MpChit35, MpChit38 and MpChit41 from *M. pulcherrima* have been assigned to the GenBank accession numbers OR941418, OR941419 and OR941420, respectively. NetNglyc 1.0 server (https://services.healthtech.dtu.dk/services/NetNGlyc-1.0/) was used to predict *N*-glycosylation sites at recombinant protein sequences (> 0.5 threshold) and NetOglyc 4.0 server to predict O-glycosylation sites (https://services.healthtech.dtu.dk/services/NetOGlyc-4.0/). Signal peptides of proteins were predicted using SignalP Version 6.0 (> 0.5 threshold) machine learning mode (https://services.healthtech.dtu.dk/services/SignalP-6.0/). Multiple sequence alignments (MSAs) were constructed and analysed within BioEdit Version 7.2 [56] and ESPrit Version 3.0 interfaces [57] Protein modeling was performed using Alpha-Fold [58] implemented at Centro de Computación Científica (UAM) and the structural 3D constructed models were analysed using UCSF Chimera Version 1.13.1 interface [59]. The quality of generated models was inferred from predicted per-residue confidence scores or pDDLT (0-100). MobiDB-lite Version 3.0. Method [60] was implemented for accurate prediction of intrinsically disordered regions in protein sequences. Theoretical molecular weight of chitinases was predicted from amino acid composition using ProtParam tool (https://web.expasy.org/protparam/) from Expasy repository. Oligonucleotide primer pairs were designed and synthesised in IDT, Newark, New Jersey, USA.

### Electronic supplementary material

Below is the link to the electronic supplementary material.

**Additional File 1.** Oligonucleotides employed in this work **(Table S1)**. Multiple sequence alignment of MpChit35-38 and homologous chitinases **(Figure S1)**. Multiple sequence alignment of MpChit41 and homologous chitinases **(Figure S2)**. Michaelis-Menten fit **(Figure S3)**. Peaks and percentage of peak intensities of the mass spectrum corresponding to the products mixtures of CC hydrolysis **(Table S2).** Peaks and percentage of peak intensities of the mass spectrum corresponding to the products mixtures of chitosan CHIT50.1 hydrolysis **(Table S3)**. Peaks and percentage of peak intensities of the mass spectrum corresponding to the products mixtures of chitosan CHIT50.1 hydrolysis **(Table S4)**.

## Data Availability

All data generated or analysed during this study are included in this manuscript.

## Abbreviations

COS: Chito-oligosaccharides
*fa*COS: Fully acetylated COS
*pa*COS: Partially acetylated COS
*fd*COS: Fully deacetylated COS
CC: Colloidal chitin
CID: Chitinase insertion domain
DP: Degree of polymerization
DA: Degree of acetylation
DD: Degree of deacetylation
PA: Pattern of acetylation
GH18: Glycosyl hydrolase family 18
gDNA: Genomic DNA
MSA: Multiple sequence analysis
GlcNAc: *N*-acetyl-D-glucosamine
GlcN: D-glucosamine
MS: Mass spectrometry
