# Supplementary material for: Chitinous material bioconversion by three new chitinases from the yeast Mestchnikowia pulcherrima
Source: Microb Cell Fact. 2024 Jan 20;23:31. doi: 10.1186/s12934-024-02300-9 (PMC10799394; doi:10.1186/s12934-024-02300-9)
Supplement: Supplementary file 1 — Additional File 1. Oligonucleotides employed in this work (Table S1). Multiple sequence alignment of MpChit35-38 and homologous chitinases (Figure S1). Multiple sequence alignment of MpChit41 and homologous chitinases (Figure S2). Michaelis-Menten fit (Figure S3). Peaks and percentage of peak intensities of the mass spectrum corresponding to the products mixtures of CC hydrolysis (Table S2). Peaks and percentage of peak intensities of the mass spectrum corresponding to the products mixtures of chitosan CHIT50.1 hydrolysis (Table S3). Peaks and percentage of peak intensities of the mass spectrum corresponding to the products mixtures of chitosan CHIT50.1 hydrolysis (Table S4). [file 12934_2024_2300_MOESM1_ESM.docx]

**Chitinous material bioconversion by three new chitinases from the yeast *Mestchnikowia pulcherrima***

Marina Minguet-Lobato^1,2^, Fadia V. Cervantes^2^, Noa Míguez^2^, Francisco J. Plou^2*^ and María Fernández-Lobato^1*^

^1^ Department of Molecular Biology, Centre for Molecular Biology Severo Ochoa (CSIC-UAM), University Autonomous from Madrid. C/ Nicolás Cabrera, 1. Cantoblanco. 28049 Madrid, Spain.

^2^ Institute of Catalysis and Petrochemistry, CSIC. C/ Marie Curie, 2. Cantoblanco. 28049 Madrid, Spain.

* Corresponding authors: Francisco J. Plou (e-mail address: [fplou@icp.csic.es](mailto:fplou@icp.csic.es)); María Fernández-Lobato (e-mail address: [mfernandez@cbm.csic.es](mailto:mfernandez@cbm.csic.es)).

| Table S1 | Oligonucleotides employed in this work. |
| --- | --- |
| Figure S1 | Multiple sequence alignment of MpChit35, MpChit38 and homologous chitinases. |
| Figure S2 | Multiple sequence alignment of MpChit41 and homologous chitinases. |
| Figure S3 | Michaelis-Menten fit. |
| Table S2 | Peaks and percentage of peak intensities of the mass spectrum corresponding to the products mixtures of colloidal chitin hydrolysis. |
| Table S3 | Peaks and percentage of peak intensities of the mass spectrum corresponding to the products mixtures produced from the chitosan CHIT50.1 hydrolysis. |
| Table S4 | Peaks and percentage of peak intensities of the mass spectrum corresponding to the products mixtures produced from the chitosan CHIT50.2 hydrolysis. |

**Table S1. Oligonucleotides employed in this work.**

| **Primer** | **Sequence (5҆-3҆)** |
| --- | --- |
| CHI41F1 | TTACTCACTCAATGGGTAGGG |
| CHI41R1 | ATGAGTCTG GGTGGACACG |
| CHI38F1 | ATGTTGATGCAACCATTTTTATG |
| CHI38R1 | TCAGACTTTGAACTTTGGCTTG |
| CHI35F1 | ATGTTGATGCCCACTATT CTC TG |
| CHI35R1 | TCAAACCTTAACAAACTT CGG C |
| CHI41F2 | GGTATCTCTCGAGAAAAGAGAGGCTGAAGCTagtctgggtggacacgtca |
| CHI41R2 | cctacccattgagtgagtaaGAATTCGAGCTCGGTACCCGGGGAT |
| CHI38F2 | gaaggggtatctctcgagaaaagagaggctgaagctATGCAGCCTCCAAAAGGAG |
| CHI38R2 | gatccccgggtaccgagctcgaattcTCAGACTTTGAACTTTGGCTTG |
| CHI35F2 | ggtatctctcgagaaaagagaggctgaagctCAGCCACCCAAAGGTGTCG |
| CHI35R2 | atccccgggtaccgagctcgaattcTCAAACCTTAACAAACTTCGGC |
| pIB4F | GAATTCGAGCTCGGTACCC |
| pIB4R | AGCTTCAGCCTCTCTTTTCTC |
| AOX1 | GACTGGTTCCAATTGACAAGC |
| AOX2 | GGATGTCAGAATGCCATTTGC |

Regions annealing with pIB4 sequences are shown in lower case.

**
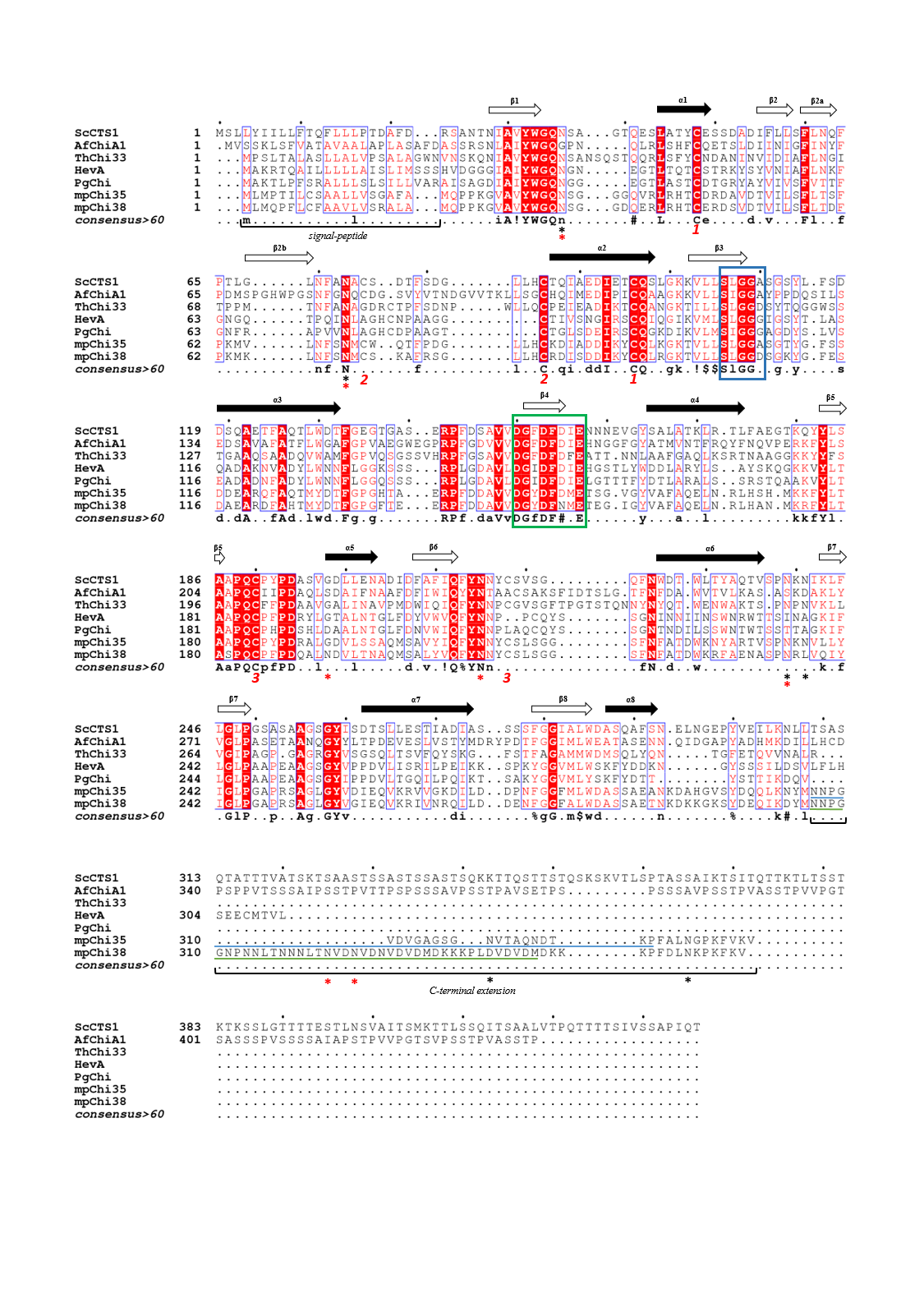
**

**Figure S1. Multiple sequence alignment of MpChit35, MpChit38 and homologous chitinases.** Sequences ScCTS1 (PDB: 2UY2), AfChiA1 (PDB: 2XTK), ThChi33 (PDB: 7ZYA), HevA (PDB: 1HVQ) and PgChi (PDB: 4TOQ) are shown. Conserved catalytic motifs are boxed in green. Conserved substrate binding motifs are boxed in blue. Conserved cysteine residue pairs are numbered in red. Predicted secondary elements are indicated. Predicted N-glycosylation sites of MpChit35 (black) and MpChit38 (red) are market with asterisks. Signal peptides and C-terminal extensions are indicated. Consensus intrinsically disorder sequences are underlined. Consensus sequence is displayed.

**
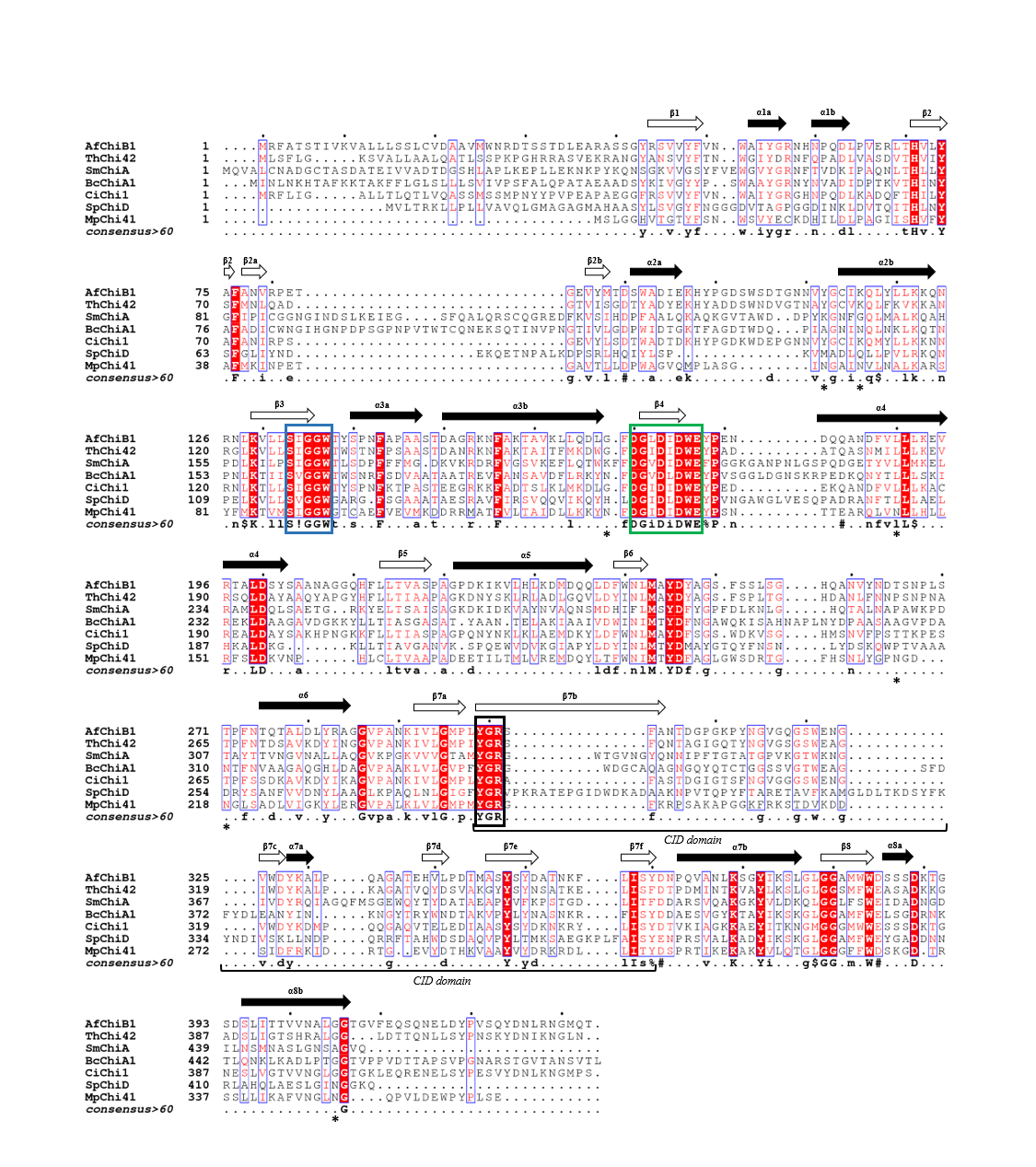
**

**Figure S2. Multiple sequence alignment of MpChit41 and homologous chitinases.** Sequences of AfChiB1 (PDB: 1W9P), ThChit42 (PDB: 6EPB), CiChi1 (PDB: 1D2K), catalytic domain of SmChiA (PDB: 5Z7M), catalytic domain of BcChiA1 (PDB: 1ITX) and SpChiD (PDB: 4NZC) are shown. GH18 conserved catalytic motifs are boxed in green. GH18 conserved substrate binding motifs of catalytic domain are boxed in blue. CID domains are indicated. GH18 conserved motifs of CID domain are boxed in black. Predicted N-glycosilation sites are market with asterisks. Consensus sequence is displayed.

**
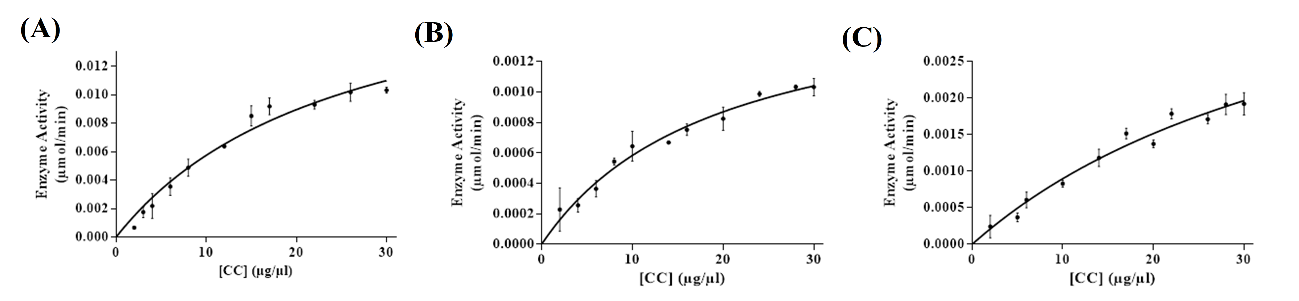
**

**Figure S3. Michaelis-Menten fit.** **(A)** MpChit35. **(B)** MpChit38. **(C)** MpChit41. Colloidal Chitin 0-30 µg µl^-1^ was used as substrate. R2 Square > 0.95. Values are the result of three independent replicates and standard errors are shown.

**Table S2. Peaks and percentage of peak intensities of the mass spectrum corresponding to the products mixtures of the colloidal chitin hydrolysis.**

|  | | **Intensity (%)** | | |
| --- | --- | --- | --- | --- |
| **m/z** | **Corresponding COS** | **MpChit35** | **MpChit38** | **MpChit41** |
| 204.039 | [(GlcNAc) – H_2_O + H]^+^ | 59.9 | 13.1 | 5.5 |
| 447.195 | [(GlcNAc)_2_ + Na]^+^ | **100.0** | 36.4 | 39.8 |
| 463.173402 | [(GlcNAc)-(GlcN) + K]^+^ | 10.1 | 2.7 | 3.7 |
| 608.271177 | [(GlcNAc)_2_-(GlcN) + Na]^+^ | 4.0 | 2.1 | 1.9 |
| 650.283268 | [(GlcNAc)_3_ + Na]^+^ | 47.1 | **100.0** | **100.0** |
| 785.222082 | [(GlcNAc)_2_-(GlcN)_2_ + K]^+^ | 6.8 | 1.0 | 1.2 |
| 811.339375 | [(GlcNAc)_3_-(GlcN) + Na]^+^ | 5.1 | 4.2 | 4.7 |
| 853.341325 | [(GlcNAc)_4_ + Na]^+^ | 1.3 | 40.0 | 29.1 |
| 946.266249 | [(GlcNAc)_2_-(GlcN)_3_ + K]^+^ | 0.5 | - | - |
| 988.269724 | [(GlcNAc)_3_-(GlcN)_2_ + K]^+^ | 2.8 | 2.5 | 2.4 |
| 1014.38297 | [(GlcNAc)_4_-(GlcN) + Na]^+^ | 0.3 | 1.7 | 2.0 |
| 1056.39519 | [(GlcNAc)_5_ + Na]^+^ | 0.9 | 1.7 | 1.2 |
| 1149.31684 | [(GlcNAc)_3_-(GlcN)_3_ + K]^+^ | 0.3 | - | - |
| 1175.34689 | [(GlcNAc)_4_-(GlcN)_2_ + Na]^+^ | 0.3 | - | 0.6 |
| 1191.32368 | [(GlcNAc)_4_-(GlcN)_2_ + K]^+^ | - | 0.5 | 0.4 |
| 1217.4396 | [(GlcNAc)_5_-(GlcN) + Na]^+^ | 0.3 | - | 0.4 |
| 1259.44037 | [(GlcNAc)_6_ + Na]^+^ | 0.6 | 1.0 | 0.9 |
| 1462.48237 | [(GlcNAc)_7_ + Na]^+^ | 0.5 | 0.9 | 0.7 |
| 1665.52584 | [(GlcNAc)_8_ + Na]^+^ | 0.2 | 0.6 | 0.4 |

[M+H]^+^, [M+Na]^+^ and [M+K]^+^ peaks were detected in positive mode.

**Table S3. Peaks and percentage of peak intensities of the mass spectrum corresponding to the products mixtures obtained from the CHIT50.1 hydrolysis.**

|  | | **Intensity (%)** | | |
| --- | --- | --- | --- | --- |
| **m/z** | **Corresponding COS** | **MpChit35** | **MpChit38** | **MpChit41** |
| 204.050058 | [(GlcNAc) – H_2_O + H]^+^ | 16.6 | 7.5 | 29.4 |
| 405.21023 | [(GlcNAc)-(GlcN) + Na]^+^ | 2.8 | 2.9 | - |
| 447.197855 | [(GlcNAc)_2_ + Na]+ | 24.2 | 55.7 | 23.3 |
| 608.281978 | [(GlcNAc)_2_-(GlcN) + Na]^+^ | **100.0** | **100.0** | **100.0** |
| 650.300282 | [(GlcNAc)_3_ + Na]+ | 3.8 | 99.0 | 99.0 |
| 769.351073 | [(GlcNAc)_2_-(GlcN)_2_ + Na]^+^ | 6.3 | 0.7 | - |
| 811.354954 | [(GlcNAc)_3_-(GlcN) + Na]^+^ | 11.0 | 28.1 | 22.0 |
| 853.35284 | [(GlcNAc)_4_] + Na]+ | - | 10.4 | 16.4 |
| 946.305175 | [(GlcNAc)_2_-(GlcN)_3_ + K]^+^ | 3.0 | 1.8 | - |
| 972.411091 | [(GlcNAc)_3_-(GlcN)_2_ + Na]^+^ | 6.1 | 0.3 | - |
| 1014.40114 | [(GlcNAc)_4_-(GlcN) + Na]^+^ | - | 14.9 | 6.4 |
| 1091.45098 | [(GlcNAc)_2_-(GlcN)_4_ + Na]^+^ | 6.1 | - | - |
| 1133.45629 | [(GlcNAc)_3_-(GlcN)_3_ + Na]^+^ | 29.7 | - | - |
| 1175.4591 | [(GlcNAc)_4_-(GlcN)_2_ + Na]^+^ | 1.4 | 0.8 | - |
| 1217.47286 | [(GlcNAc)_5_-(GlcN) + Na]^+^ | - | 0.8 | - |
| 1252.48648 | [(GlcNAc)_5_-(GlcN)_2_ + Na]^+^ | 0.8 | - | - |
| 1294.50619 | [(GlcNAc)_3_-(GlcN)_4_ + Na]_+_ | 4.2 | - | - |
| 1336.51208 | [(GlcNAc)_4_-(GlcN)_3_ + Na]^+^ | 2.3 | - | - |
| 1413.55809 | [(GlcNAc)_2_-(GlcN)_6_ + Na]^+^ | 0.5 | - | 3.1 |
| 1455.54893 | [(GlcNAc)_3_-(GlcN)_5_ + Na]^+^ | 3.3 | - | - |
| 1497.55282 | [(GlcNAc)_4_-(GlcN)_4_ + Na]^+^ | 2.5 | - | - |
| 1574.5982 | [(GlcNAc)_2_-(GlcN)_7_ + Na]^+^ | 0.9 | - | 3.5 |
| 1616.60876 | [(GlcNAc)_3_-(GlcN)_6_ + Na]^+^ | 5.9 | - | 4.8 |
| 1658.61325 | [(GlcNAc)_4_-(GlcN)_5_ + Na]^+^ | 8.8 | - | 5.6 |
| 1700.58748 | [(GlcNAc)_5_-(GlcN)_4_ + Na]^+^ | 0.4 | - | 2.1 |
| 1735.64292 | [(GlcNAc)_2_-(GlcN)_8_ + Na]^+^ | 0.3 | - | 2.7 |
| 1777.66553 | [(GlcNAc)_3_-(GlcN)_7_ + Na]^+^ | 0.9 | - | 2.3 |
| 1819.66593 | [(GlcNAc)_4_-(GlcN)_6_ + Na]^+^ | 1.4 | - | 1.9 |
| 1861.68435 | [(GlcNAc)_5_-(GlcN)_5_ + Na]^+^ | 0.5 | - | 1.9 |
| 1938.72276 | [(GlcNAc)_3_-(GlcN)_8_ + Na]^+^ | 0.6 | - | 2.7 |
| 1980.73596 | [(GlcNAc)_4_-(GlcN)_7_ + Na]^+^ | 1.1 | - | 2.3 |
| 2057.78343 | [(GlcNAc)_2_-(GlcN)_10_ + Na]^+^ | 0.4 | - | 2.3 |
| 2099.79846 | [(GlcNAc)_3_-(GlcN)_9_ + Na]^+^ | 0.9 | - | 4.0 |
| 2141.81443 | [(GlcNAc)_4_-(GlcN)_8_ + Na]^+^ | 2.0 | - | 5.4 |
| 2260.87614 | [(GlcNAc)_3_-(GlcN)_10_ + Na]^+^ | 0.3 | - | 3.3 |
| 2302.88329 | [(GlcNAc)_4_-(GlcN)_9_ + Na]^+^ | 0.4 | - | 1.9 |
| 2395.95165 | [(GlcNAc)_2_-(GlcN)_12_ + Na]^+^ | - | - | 1.5 |
| 2625.08418 | [(GlcNAc)_4_-(GlcN)_11_ + Na]^+^ | 0.3 | - | 1.5 |

[M+H]^+^, [M+Na]^+^ and [M+K]^+^ peaks were detected in positive mode.

**Table S4. Peaks and percentage of peak intensities of the mass spectrum corresponding to the products mixtures produced from the CHIT50.2 hydrolysis.**

|  | | **Intensity (%)** | | |
| --- | --- | --- | --- | --- |
| **m/z** | **Corresponding COS** | **MpChit35** | **MpChit38** | **MpChit41** |
| 204.039013 | [(GlcNAc) – H_2_O + H]^+^ | 18.0 | 13.8 | 6.4 |
| 405.170122 | [(GlcNAc)-(GlcN) + Na]^+^ | 2.3 | 3.7 | 1.1 |
| 447.179338 | [(GlcNAc)_2_ + Na]+ | 21.1 | 27.8 | 25.4 |
| 566.233963 | [(GlcNAc)-(GlcN)_2_ + Na]^+^ | 2.2 | - | - |
| 608.242376 | [(GlcNAc)_2_ -(GlcN) + Na]^+^ | **100.0** | **100.0** | **100.0** |
| 650.284309 | [(GlcNAc)_3_ + Na]+ | 2.8 | 29.7 | 47.5 |
| 769.300813 | [(GlcNAc)_2_ -(GlcN)_2_ + Na]+ | 7.4 | 2.3 | - |
| 785.252787 | [(GlcNAc)_2_ -(GlcN)_2_ + K]^+^ | 0.5 | 0.9 | 1.7 |
| 811.308766 | [(GlcNAc)_3_-(GlcN) + Na]^+^ | 10.4 | 21.4 | 26.2 |
| 853.325011 | [(GlcNAc)_4_ + Na]+ | - | 8.4 | 8.1 |
| 930.360229 | [(GlcNAc)_2_-(GlcN)_3_ + Na]^+^ | 3.0 | 0.8 | 0.6 |
| 946.2625 | [(GlcNAc)_2_-(GlcN)_3_ + K]^+^ | 1.6 | 3.3 | 8.3 |
| 972.347894 | [(GlcNAc)_3_-(GlcN)_2_ + Na]^+^ | 7.4 | 9.9 | 3.5 |
| 1014,39779 | [(GlcNAc)_4_-(GlcN) + Na]^+^ | - | 9.1 | 8.3 |
| 1091.38723 | [(GlcNAc)_2_-(GlcN)_4_ + Na]^+^ | 9.0 | - | 1.2 |
| 1133.39347 | [(GlcNAc)_3_-(GlcN)_3_ + Na]^+^ | 39.5 | 21.1 | 13.0 |
| 1175.43813 | [(GlcNAc)_4_-(GlcN)_2_ + Na]^+^ | - | 27.8 | 2.3 |
| 1217.42523 | [(GlcNAc)_5_-(GlcN)_5_ + Na]^+^ | - | 0.5 | - |
| 1252.42734 | [(GlcNAc)_2_-(GlcN)_5_ + Na]^+^ | 1.2 | - | 0.7 |
| 1294.4254 | [(GlcNAc)_3_-(GlcN)_4_ + Na]^+^ | 5.8 | 1.7 | 1.1 |
| 1336.43761 | [(GlcNAc)_4_-(GlcN)_3_ + Na]^+^ | 2.7 | 7.3 | 2.1 |
| 1371.52367 | [(GlcNAc)-(GlcN)_7_ + Na]^+^ | - | - | 0.5 |
| 1378.4519 | [(GlcNAc)_5_-(GlcN)_2_ + Na]^+^ | 0.2 | 1.0 | - |
| 1413.46595 | [(GlcNAc)_2_-(GlcN)_6_ + Na]^+^ | 0.8 | - | 0.8 |
| 1455.4702 | [(GlcNAc)_3_-(GlcN)_5_ + Na]^+^ | 4.4 | 1.1 | 1.4 |
| 1497.47171 | [(GlcNAc)_4_-(GlcN)_4_ + Na]^+^ | 3.1 | 4.8 | 1.7 |
| 1532.56174 | [(GlcNAc)-(GlcN)_8_ + Na]^+^ | - | - | 0.6 |
| 1539.47112 | [(GlcNAc)_5_-(GlcN)_3_ + Na]^+^ | 0.3 | 3.0 | 0.9 |
| 1559.64511 | [(GlcNAc)_6_-(GlcN)_2_ + H]^+^ | - | - | 0.6 |
| 1574.5073 | [(GlcNAc)_2_-(GlcN)_7_ + Na]^+^ | 1.1 | 0.4 | 2.2 |
| 1616.51276 | [(GlcNAc)_3_-(GlcN)_6_ + Na]^+^ | 9.0 | 1.5 | 4.8 |
| 1636.54953 | [(GlcNAc)_4_-(GlcN)_5_ + H]^+^ | 1.0 | 0.7 | 0.6 |
| 1658.51514 | [(GlcNAc)_4_-(GlcN)_5_ + Na]^+^ | 13.0 | - | 10.1 |
| 1700.52917 | [(GlcNAc)_5_-(GlcN)_4_ + Na]^+^ | 0.4 | 25.4 | 9.8 |
| 1735.55549 | [(GlcNAc)_2_-(GlcN)_8_ + Na]^+^ | 0.5 | 0.4 | 1.1 |
| 1742.55849 | [(GlcNAc)_6_-(GlcN)_3_ + Na]^+^ | - | 0.8 | - |
| 1777.56219 | [(GlcNAc)_3_-(GlcN)_7_ + Na]^+^ | 1.3 | 0.6 | 1.9 |
| 1819.5741 | [(GlcNAc)_4_-(GlcN)_6_ + Na]^+^ | 2.8 | 1.2 | 1.8 |
| 1861.57141 | [(GlcNAc)_5_-(GlcN)_5_ + Na]^+^ | 0.8 | 2.7 | 1.3 |
| 1896.69635 | [(GlcNAc)_2_-(GlcN)_9_ + Na]^+^ | - | - | 0.6 |
| 1903.66189 | [(GlcNAc)_6_-(GlcN)_4_ + Na]^+^ | - | 1.5 | 0.6 |
| 1938.61385 | [(GlcNAc)_3_-(GlcN)_8_ + Na]^+^ | 1.3 | 0.6 | 1.6 |
| 1980.63004 | [(GlcNAc)_4_-(GlcN)_7_ + Na]^+^ | 1.7 | 0.9 | 1.9 |
| 2057.66312 | [(GlcNAc)_2_-(GlcN)_10_ + Na]^+^ | 0.6 | 0.3 | 0.9 |
| 2064.72442 | [(GlcNAc)_6_-(GlcN)_5_ + Na]^+^ | - | 1.3 | 0.7 |
| 2099.67993 | [(GlcNAc)_3_-(GlcN)_9_ + Na]^+^ | 1.4 | 0.4 | 2.2 |
| 2141.69677 | [(GlcNAc)_4_-(GlcN)_8_ + Na]^+^ | 4.4 | 1.4 | 3.7 |
| 2260.75043 | [(GlcNAc)_3_-(GlcN)_10_ + Na]^+^ | 0.6 | - | 0.8 |
| 2302.76748 | [(GlcNAc)_4_-(GlcN)_9_ + Na]^+^ | 0.9 | 0.5 | 1.1 |
| 2463.8326 | [(GlcNAc)_4_-(GlcN)_10_ + Na]^+^ | 0.4 | 0.3 | 0.5 |

[M+H]^+^, [M+Na]^+^ and [M+K]^+^ peaks were detected in positive mode.
